# Factors that shape pregnant women’s perceptions regarding the safety of cannabis use during pregnancy

**DOI:** 10.1186/s42238-022-00128-x

**Published:** 2022-04-06

**Authors:** Mohamed A. Satti, Eda G. Reed, Elizabeth S. Wenker, Stephanie L. Mitchell, Jay Schulkin, Michael L. Power, A. Dhanya Mackeen

**Affiliations:** 1 Department of Obstetrics and Gynecology, Division of Maternal-Fetal Medicine, Geisinger, Danville, USA; 2 Center for Species Survival, Smithsonian National Zoological Park and Conservation Biology Institute, 3001 Connecticut Ave NW, Washington DC, 20008 USA; 3 School of Public Health, University of California, Berkeley, Berkeley, USA; 4 Obstetrics & Gynecology, University of Washington School of Medicine, Seattle, USA

**Keywords:** Cannabis, Alcohol, Tobacco, Safety, Pregnancy, Attitudes

## Abstract

**Background:**

Cannabis use among pregnant women has increased. We surveyed pregnant women in rural Pennsylvania to examine cannabis use and opinions regarding its safety during pregnancy. We examined associations between challenges of pregnancy (e.g., exhaustion, pain, nausea) and cannabis use.

**Methods:**

A cross-sectional survey was administered to a convenience sample of English-speaking pregnant women receiving prenatal care at Geisinger, May–June 2019. Principal component analysis (PCA) was used to construct three scores (overwhelmed/exhausted, happy/optimistic, and health worries) based on 10 questions regarding common experiences during pregnancy (e.g., nausea/vomiting, pain, exhaustion, mood). A score based on four questions regarding cannabis safety during pregnancy was also constructed.

**Results:**

From a maximum of 300 surveys distributed, 284 were completed (95%). Most participants were white (87%), married (49%) or living with a partner (38%), and had private health insurance (62%). Most women indicated it was unsafe to use alcohol and tobacco products during pregnancy (> 90%), but that proportion dropped to 82% and 63% regarding recreational cannabis and medical cannabis, respectively. Only women with prior cannabis use (23% of sample) continued to do so during pregnancy: 57% of women reporting daily cannabis use prior to pregnancy continued to use cannabis during pregnancy with 33% reporting daily use. Two thirds of users during pregnancy indicated they were self-medicating for: nausea (90%), anxiety (70%), insomnia (30%), and pain management (30%). Many (56%) of the women who used cannabis during pregnancy believed it is safe. Younger women and women who were overwhelmed/exhausted or less happy/optimistic were more likely to believe cannabis use is safe. Women valued healthcare provider advice more than advice from family and friends. Study strengths include a high response rate. Weaknesses include self-report and that is was a convenience sample; however, the demographics of the sample were similar to past studies.

**Conclusion:**

Women with a history of cannabis use, especially daily use, are at risk of continuing during pregnancy and should receive counseling. Younger women and women with greater stressors during pregnancy also are at greater risk. Screening for prior use and for stressors may identify patients that would benefit from enhanced counseling.

## Background

The social acceptance of cannabis products in the United States (US) has increased substantially in the last half decade (Hasin et al. 2019; Brown et al. 2017). Legalization of cannabis in many states resulted in a boom in the commercialized cannabis industry. The expanding cannabis commerce has led to an increase in cannabis dispensaries across many states. The growth in cannabis dispensary businesses has increased access to and awareness of cannabis products and their legitimate uses. In non-pregnant populations, the odds of cannabis use increased in states upon the passing of recreational cannabis laws. This increase in cannabis use was most evident among Hispanic and non-Hispanic whites; notably, legalization of cannabis did not contribute to an increase in cannabis use among non-Hispanic Black individuals (Martins et al. 2021). It also appears to have led to an increase in cannabis use during pregnancy, with potential public health implications (Cooke et al. 2018). In non-pregnant populations, increases in cannabis use are documented with the onset of the COVID-19 pandemic with the highest increases in use occurring in large metropolitan areas. Medicinal legal status of cannabis has been associated with higher usage of cannabis among nonmetropolitan users. Additionally, uninsured adults residing in rural areas exhibited higher usage of cannabis when compared to insured adults residing in rural areas (Moore et al. 2021).

The prevalence of reported cannabis use during pregnancy increased 62% from 2002 to 2014 (Brown et al. 2017; Skelton et al. 2021). Reasons for cannabis use during pregnancy include some of the common challenges of pregnancy, such as nausea/vomiting and to improve mood (Chang et al. 2019; Vanstone et al. 2021; Ko et al. 2020). Increased access to legal cannabis products may influence a pregnant woman’s judgment regarding the safety of cannabis and could result in continued or increased use during pregnancy (Bayrampour et al. 2019; Mark et al. 2017). Pregnant women’s attitudes and beliefs towards cannabis use during pregnancy may be influenced by the legal status of cannabis in that state. For example, in a survey administered at a regional perinatal center in New Jersey, more than 90% of the pregnant women reported that legalization of cannabis would increase their likelihood of use (Ng et al. 2020). Cannabis use during pregnancy significantly increased in several western US states after decriminalization laws were passed in those states (Grant et al. 2018; Gnofam et al. 2020; Skelton et al. 2020).

In this study, we asked pregnant women in a largely rural, white patient population about their cannabis use, how they view cannabis use during pregnancy, and the role their healthcare providers play in counseling about use. We compared their opinions about cannabis to their opinions regarding alcohol and tobacco use, substances which are widely understood to be harmful to pregnant women and their fetuses. We assessed pregnant women’s experiences of various challenges during pregnancy (e.g., exhaustion, mood, nausea/vomiting and sleep issues) and their perception of advice from family, friends, and healthcare providers regarding managing these challenges. We combined these data to assess the relative opinion of cannabis/tetrahydrocannabinol (THC) use during pregnancy and to identify factors that were associated with belief that cannabis is safe to use during pregnancy. This information could be useful to inform clinicians regarding patients that could benefit from enhanced counseling regarding cannabis use during pregnancy.

## Methods

The study was conducted at two clinics at Geisinger Medical Center, a tertiary care center in Danville, Pennsylvania, that is part of a large integrated healthcare system in central and northeastern Pennsylvania. Geisinger’s patient population is largely non-Hispanic white (mean = 94.5% for pregnant women between 2006 and 2015; Power et al. 2018) and rural. A cross-sectional survey study was approved by the Geisinger Institutional Review Board (IRB #2019-0158). All pregnant women who received prenatal care at Geisinger between May 1, 2019, and June 30, 2019, were candidates for the study. During a prenatal visit, patients were provided with a study information sheet which served to obtain informed consent and a survey by clinic staff. Those who chose to participate in the study returned completed questionnaires in sealed envelopes to clinic staff. Because the information sheet was only in English, patients with poor English proficiency, defined as requiring an interpreter at their visit, had to be excluded. Exclusions and refusals were not tracked, as the IRB required complete anonymity.

The survey consisted of demographic questions and questions to assess patient perspectives on various challenges during pregnancy, including pain, insomnia, nausea/vomiting, and substance use. Participants were asked to respond to questions based on how they felt during the week prior to the survey. Questions were kept in a consistent format to aid in comparisons and the development of scores for statistical analysis. Patient medical records were not accessed and Protected Health Information was not requested, except for year of birth. Surveys took approximately 10 minutes to complete. Subjects were not compensated.

Data were analyzed using SPSS (version 28.0, IBM, NY). Categorical data were analyzed using chi-square tests. An opinion score regarding the safety of cannabis use during pregnancy was constructed by averaging the responses from four questions about the safety of smoking or consuming edible cannabis/THC products based on a scale of 1 = strongly agree, 2 = agree, 3 = disagree, and 4 = strongly disagree it is safe (Table 1).

**Table 1:** Questions used to create the opinion score

	Strongly agree	Agree	Disagree	Strongly disagree	Do not know	Did not answer
If I have been prescribed medical marijuana, it is safe to continue during pregnancy	7 (2.5%)	19 (6.7%)	59 (20.8%)	120 (42.3%)	73(25.7%)	6 (2.1%)
Smoking marijuana in moderation is safe during pregnancy	4 (1.4%)	15 (5.3%)	48 (16.9%)	183 (64.4%)	28 (9.9%)	6 (2.1%)
THC/marijuana-based vaping is safe during pregnancy	2 (0.7%)	9 (3.2%)	51 (18.%)	190 (66.9%)	26 (9.2%)	6 (2.1%)
Edible marijuana products in moderation are safe to use during pregnancy	5 (1.8%)	13 (4.6%)	48 (16.9%)	180 (63.4%)	32 (11.3%)	6 (2.1%)

The opinion score was constructed by averaging the responses from these four questions about the safety of smoking or consuming edible marijuana/THC products based on a scale of 1 = strongly agree, 2 = agree, 3 = disagree, and 4 = strongly disagree it is safe

Principal component analysis (PCA) reduced answers from 10 questions on the women’s experiences to scores on three axes (eigenvalues greater than 1.0): overwhelmed/exhausted, happy/optimistic, and health worries (positive values indicate more likely to experience the feeling; Table 2). Health worries included being worried about healthy eating, gaining too much weight and nausea/vomiting during pregnancy (Table 2). The Kaiser-Meyer-Olkin Measure of Sampling Adequacy and Bartlett’s test of Sphericity were used to assess the appropriateness of using PCA to create these scores. Scores for different groups were compared using one-way analysis of variance and correlation was used to assess relationships between other parameters. Statistical significance was designated as *p* < 0.05.

**Table 2:** Data and results for the scores derived by principal component analysis (PCA)

	Factor loadings	Every day	Most days	A few times	Never
Overwhelmed/exhausted (21.4% of variance)					
Had difficulty sleeping	0.799	16.0%	27.3%	43.6%	13.1%
Felt exhausted	0.762	21.0%	34.9%	41.5%	2.6%
I was in pain	0.639	6.2%	7.7%	42.3%	43.8%
Felt overwhelmed	0.631	9.0%	22.7%	50.5%	17.7%
Happy/optimistic (18.8% of variance)					
Felt optimistic and hopeful	0.843	28.0%	50.9%	17.8%	3.3%
Felt safe and relaxed	0.748	53.8%	36.7%	8.7%	0.7%
Felt happy and excited	0.692	32.1%	51.3%	14.8%	1.8%
Health concerns (15.3% of variance)					
Worried about being able to eat healthy	0.694	8.3%	11.6%	31.2%	48.9%
Worried about gaining too much weight	0.686	11.8%	12.3%	35.7%	40.4%
Nausea and/or vomiting	0.666	5.1%	10.1%	42.8%	42.0%

The PCA used the answers to: “During the week before this day how often did you experience the following?” Three axes with eigenvalues above 1.0 were determined that explained 55.5% of the variance. The Kaiser-Meyer-Olkin Measure of Sampling Adequacy was 0.691, indicating moderate adequacy; Bartlett’s test of Sphericity was 469.6. df = 45, *P* < 0.001. Percentages given exclude women who did not answer the question

## Results

Of the 300 distributed surveys, 284 were returned for a response rate of 95%. Most women were white (87%), married or living with a partner (88%), and had private insurance (62%) with a median age of 29 years (range 16–43 years). Most of the women in this survey (93%) had achieved at least a high school education; 42% graduated college. Median gestational age at time of survey completion was 27 weeks (range 9–39 weeks). For one third of the women, this was their first pregnancy. Complete demographic data are presented in Table 3.

**Table 3:** Demographics of the 284 pregnant women who returned surveys

Age in years; median (range)	29 (16–43)
Race:	
White	246 (86.6%)
African American	15 (5.3%)
Other	20 (7.0%)
Did not answer	3 (1.1%)
Relationship status:	
Married	140 (49.3%)
Not married but living with partner	109 (38.4%)
In a relationship but living with partner	19 (6.7%)
Not in a relationship	15 (5.8%)
Did not answer	1 (0.4%)
Education:	
Less than high school diploma	14 (4.9%)
High school diploma	78 (27.5%)
Some college	68 (23.9%)
College graduate	80 (28.2%)
Graduate or professional degree	39 (13.7%)
Did not answer	5 (1.8%)
Insurance status:	
Private insurance	176 (62.0%)
Medicaid	91 (32.0%)
No insurance	12 (4.2%)
Did not answer	5 (1.8%)
Received majority of their prenatal care from:	
Obstetrician	134 (47.2%)
Certified nurse midwife	40 (14.1%)
Nurse practitioner or physician’s assistant	97 (34.2%)
Did not answer	13 (4.6%)

Almost all respondents believed that alcohol consumption (95%) and tobacco products were not safe to use during pregnancy: 97% felt smoking was not safe, 93% vaping, 90% hookah, and 95% chewing tobacco. Most (91%) also indicated that second-hand smoke was bad for the baby. However, only 10% strongly disagreed that smoking tobacco should be avoided during pregnancy.

A majority of women (69%) were aware that *recreational* cannabis use is not legal in Pennsylvania (29% were unsure), and about half (49%) were aware that *medical* cannabis is legal. About four-in-ten women (42%) reported that cannabis/THC containing products are easy to obtain.

For the PCA, the three axes with eigenvalues above 1.0 (overwhelmed/exhausted, happy/optimistic, and health worries) explained 55.5% of the variance. The Kaiser-Meyer-Olkin Measure of Sampling Adequacy was 0.691, indicating moderate sampling adequacy; Bartlett’s test of Sphericity was 469.6. df = 45, *P* < 0.001. Thus, the three scores were deemed acceptable for further analyses.

Generally, respondents felt that using cannabis during pregnancy was not safe. The median cannabis safety score was 3.7, and 74.7% of the women had a score of 3.0 or higher (1 = strongly agree that cannabis is safe and 4 = strongly disagree that cannabis is safe). The cannabis safety score was positively associated with age (*r* = 0.310, *P* < 0.001) and with the overwhelmed/exhausted factor score (*r* = 0.189, *P* = 0.002) and negatively associated with the happy/optimistic score (*r* = − 0.272, *P* < 0.001).

When the three PCA axes scores were considered, women further into their pregnancy were more likely to report feeling overwhelmed/exhausted (*r* = 0.297, *P* < 0.001) and less likely to report health worries (*r* = − 0.161, *P* = 0.009), but there was no effect on feeling happy/optimistic (*r* = − 0.082, *P* = 0.183). The lower rate of health worries in women at later gestation was driven by a reduction in experiencing nausea/vomiting (*r* = − 0.156, *P* = 0.009). Married women were less likely to report feeling overwhelmed/exhausted (− 0.164 ± 0.083 versus 0.165 ± 0.089, *P* = 0.007) and more likely to report being happy/optimistic (0.177 ± 0.079 versus − 0.180 ± 0.091, *P* = 0.003) as compared to unmarried women.

Many of the women (44%) reported using medications to treat nausea, pain, anxiety, and/or insomnia during their pregnancy, and 7.9% of those women reported self-medicating with cannabis/THC to address one of these conditions (Table 4.) Pregnant women who required medication were more likely (scored higher) to be overwhelmed/exhausted and have health concerns but did not differ on the happy/optimistic axis (data not shown). About one-in-five women reported either being prescribed medication for pain (18%) or self-medicating with cannabis/THC for pain (2%). Six percent of women indicated they experienced pain every day in the week prior to taking the survey, and 7% reported they felt pain most days that week. Most of the women (80%) indicated it is very important to receive advice from a medical provider regarding safe use of pain medication during pregnancy.

**Table 4:** Reasons women disclosed for self-medication with alcohol/tobacco/marijuana/other drugs in pregnancy. Many women selected more than one reason

	Yes	No	Did not answer	Indicated used cannabis product
Nausea and vomiting	11 (3.9%)	271 (95.4%)	2 (0.7%)	9
Anxiety	10 (3.5%)	273 (97.9%)	1 (0.4%)	7
Pain	7 (2.5%)	276 (97.2%)	1 (0.4%)	3
Inability to sleep	5 (1.8%)	278 (97.9%)	1 (0.4%)	3
Total	18 (6.3%)	264 (93.0%)	2 (0.7%)	10

Almost one-in-four of the participants (64, 23%) used cannabis/THC containing products prior to pregnancy, with 21 (8%) reporting they used it daily. Married women were least likely to report prior use (12% versus 36%, *P* < 0.001), and women on Medicaid were more likely to report prior use (34% versus 19%, *P* = 0.010). No women reported they began using cannabis/THC containing products for the first-time during pregnancy. Of the 64 that used cannabis prior to pregnancy, 15 (23%) continued to use it during pregnancy (5% of all respondents). Of the 21 who reported using cannabis/THC containing products daily before pregnancy, 12 (57%) continued to use cannabis products during pregnancy, with seven reporting daily use. Of the 15 women who used cannabis products during pregnancy, five offered no reason for cannabis product use. Reasons for self-intake of cannabis products during pregnancy included nausea, anxiety, pain management, and insomnia (Table 4). A majority of women who used cannabis products during pregnancy (60%) believed it is safe to smoke cannabis in moderation during pregnancy compared to 8% who used cannabis prior to, but not during pregnancy and 3% of participants who never used cannabis (*P* < 0.001). The women who continued to use cannabis while pregnant generally reported cannabis use of all kinds to be safe (opinion score 2.84 ± 0.31 versus 3.93 ± 0.14 and 4.51 ± 0.05 for women who quit using or never used, respectively, *P* < 0.001).

More than twice the number of women (61%) indicated it is very important for their provider to give them advice on the safety of cannabis product use during pregnancy compared to women who rated advice from family and friends “very important” (28%; *P* < 0.001). Almost all women who indicated advice from family and friends was very important also rated advice from their provider as important (89%) with only 3% of women valuing advice of their family and friends over their provider. The proportion of women who selected provider advice as “very important” was highest among women with prior use of cannabis products who did not ceased cannabis product usage during their current pregnancy (75% compared to 64% of women without prior use and 57% of women who used cannabis during their current pregnancy, *P* = 0.016). The proportion of women without prior use of cannabis products who reported provider advice not to be important (29%) was roughly double that for women with prior use (16% for prior use but not in pregnancy and 14% for women who used cannabis products during the current pregnancy).

## Discussion

Cannabis is used by a relatively small percentage of pregnant women in the US (range of 3.4–7.0% (Brown et al. 2017)). The proportion of women using cannabis in our study (5.3%) is in the middle of the national range. Recent studies have shown that the incidence of cannabis use has steadily increased (Brown et al. 2017; Volkow et al. 2019; Young-Wolff et al. 2019). Cannabis use during pregnancy is higher among younger mothers (El Marroun et al. 2018), consistent with the association found in our study between younger age and greater agreement that cannabis is safe during pregnancy.

Pregnant women in Colorado (Burks et al. 2016) surveyed on their attitudes and beliefs about cannabis use in pregnancy reported that cannabis use helped with stress (54%), anxiety (40%), pain (58%), and believed that it was a healthier alternative to smoking cigarettes (41%). Interviews with 25 women who used cannabis during their pregnancy found that the women generally believed that cannabis helped with nausea and appetite changes during pregnancy and improved mood. They also described cannabis as “natural” and “safe” compared to alcohol, tobacco, and prescribed medications (Chang et al. 2019). Consistent with those prior results, our study found associations between being overwhelmed/exhausted and reduced happiness/optimism with greater agreement regarding the safety of cannabis; many of the women who self-medicated with cannabis during pregnancy gave anxiety and pain management as reasons.

Studies have shown that pregnant women’s perception of risks of using cannabis during pregnancy differ between women who used cannabis prenatally and women who quit using cannabis when they discovered they were pregnant. When surveyed, 76% of women who stopped using cannabis upon finding out they were pregnant believed that cannabis was harmful to the fetus (Bayrampour et al. 2019). Women who continued with cannabis use throughout pregnancy had lower perceptions of harm with only 26% of surveyed women believing that cannabis was harmful to the fetus (Bayrampour et al. 2019). Our study also found that women who continued to use cannabis while pregnant generally considered it safe to do so.

Pregnancy can be a stressful period often associated with mixed feelings. Advice from a pregnant women’s support system (family, friends and healthcare providers) can be an important source of information and comfort. Counseling on the potential dangers of cannabis use might affect women’s perceptions of the risk, assuming the women trust their provider. Geisinger has a unique relationship with the community in that it is perceived by community members with high regard as a medical authority delivered by compassionate providers. Therefore, it was not unexpected to see that the women in this study valued advice from their healthcare provider. Although it is not surprising that a significant proportion of women who did not use cannabis before pregnancy considered advice on cannabis use from their healthcare provider unimportant (i.e., irrelevant to them), it is concerning that more than 14% of women with prior use did not value advice from their provider. These women may be resistant to counseling.

Identifying at risk women during the first prenatal visit may help healthcare providers to intervene promptly and appropriately and to make referrals to appropriate specialists. History of using cannabis prior to pregnancy was the greatest determining factor in shaping attitudes and beliefs towards cannabis in our study and was the most predictive of continued use during pregnancy, especially for daily users. However, unhappy and stressed/exhausted women may be at increased risk for cannabis use, as they were more likely to downplay the risks. Providers should screen patients to gauge their emotional wellbeing during pregnancy to determine if patients are overwhelmed/exhausted and predisposed to returning to cannabis product or other substance use during pregnancy based on prior use of these substances. These women may benefit from additional counseling and should be offered advice about the risks.

Studies have found that many women reported that they either did not receive counseling on cannabis use in pregnancy from their healthcare provider or that they only received counseling on legal implications, not health consequences (Bayrampour et al. 2019; Jarlenski et al. 2016) and were unsure about the consequences of cannabis use during pregnancy (Burks et al. 2016). Currently, 20 states legally mandate healthcare providers to report perinatal substance use to child protective services. Focusing prenatal cannabis education specifically on the legal mandate without addressing safety or risks of cannabis use in pregnancy does women a disservice and may lead women to disengage with their health care providers (Jarlenski et al. 2016). The lack of patient education on the health effects of cannabis products during pregnancy may be perceived as an indication of cannabis safety (Jarlenski et al. 2017) The perception that regular cannabis use has no health risks has increased three-fold from 2005 to 2015 in women of child-bearing age, including those who are pregnant (Jarlenski et al. 2017).

Health care provider counseling on use of cannabis in pregnancy is complicated by mixed evidence and should address that cannabis is not confirmed to be harmful. Although some studies found an association with low birth weight (Gunn et al. 2016), a recent study found no difference in birth weight or gestational age (Ko et al. 2018). A study of birth outcomes in the states of Colorado and Washington before and after cannabis legalization found an increase in congenital anomalies but no change in birthweight (Siega-Riz et al. 2020). As no data on cannabis exposure were available, the results from that study, while concerning, cannot attribute causation. Although the evidence regarding the effects of cannabis use during pregnancy on the fetus and long-term effects on later childhood development has been inconsistent and difficult to interpret, cannabis chemicals do cross the placenta and appear in breast milk (El Marroun et al. 2018). Studies on animal models have shown detrimental effects on fetal brain development (Hurd et al. 2019). There is appropriate concern among healthcare professionals regarding cannabis use during pregnancy because prenatal exposure may be associated with higher risk for psychopathology in childhood (Paul et al. 2021).

Our study has several limitations. To encourage honest responses, all answers were self-reported and not checked against medical records due to the anonymous nature of the survey. We only assessed cannabis use, not alcohol, tobacco, or opioid use, and therefore cannot assess whether cannabis use by these women was greater or less than for other substances nor could we assess multiple substance use. Exclusion of patients unable to complete surveys in English is an additional limitation of this study, although the rural white population Geisinger serves is overwhelmingly English speakers. We know that at one clinic there were no patients that required an interpreter, while at the other a small percentage did require one. We doubt many were excluded for this reason, however, due to the requirement that the survey administration assure the anonymity for all patients we cannot put a number on those excluded due to not being able to read the informed consent page. Because this surveyed population is largely rural and white (87% of participants identifying as white) caution in extending the findings to other populations is warranted. Strengths of the study include a 95% response rate for patients surveyed. Additionally, patients surveyed stated they placed high levels of trust on pregnancy advice given from their provider. This suggests a strong patient-provider relationship. Providers could leverage the trust in the patient-provider relationship as an opportunity to provide additional substance use education through-out the course of pregnancy.

The American College of Obstetricians and Gynecologists (ACOG) issued guidance in 2017 (American College of Obstetricians and Gynecologists 2017) to encourage obstetricians to counsel pregnant women about concerns regarding impaired fetal neurodevelopment; ACOG discouraged prescribing medical cannabis during preconception, pregnancy, and lactation. This survey suggests that women who have previously used cannabis should be targeted for additional counseling, as prior use appears to be a risk factor for use during pregnancy. Despite ACOG’s stance against cannabis use in pregnancy, legal access to cannabis products may result in a pregnant woman erroneously assuming cannabis use in pregnancy is safe, and this may result in continued use during pregnancy; this is additionally supported by uncertainty of adverse effects of continued use of cannabis in pregnancy and mixed evidence from the literature (Bayrampour et al. 2019).

Counseling women on the legal consequences of prenatal cannabis use (or any other substance) without also providing convincing evidence for the health implications may cause women to turn away from their healthcare providers rather than seek out their advice. Additionally, a lack of communication about the health implications of continued cannabis use in pregnancy may cause women to perceive cannabis use in pregnancy as a safer choice over other substances (Bayrampour et al. 2019). All pregnant women should be counseled regarding the lack of safety of cannabis use during pregnancy, that THC crosses the placenta and appears in breast milk and may be associated with impaired fetal/infant neurodevelopment. Research with long-term follow-up is needed to provide better guidance regarding the risks of prenatal cannabis use.

## Conclusion

The pregnant women in this largely white population were aware that tobacco and alcohol use during pregnancy was not safe but were less sure about cannabis use. About one-in-four used cannabis/THC containing products prior to pregnancy. Women with a history of cannabis use, especially daily use, are at risk of continuing during pregnancy and should receive counseling. Younger women and women with greater stressors during pregnancy are at greater risk of using cannabis. Women generally value advice from their healthcare provider, but it was concerning that a significant proportion of women who used cannabis prior to pregnancy were not counseled about the dangers of continued use during pregnancy. This suggests a need for provider education and training.

## Data Availability

The datasets used and/or analyzed during the current study are available from the corresponding author on reasonable request.

## Abbreviations

THC: Tetrahydrocannabinol
PCA: Principal component analysis
ACOG: American College of Obstetricians and Gynecologists
